# Pathway-Based Genome-Wide Association Studies for Plasma Triglycerides in Obese Females and Normal-Weight Controls

**DOI:** 10.1371/journal.pone.0134923

**Published:** 2015-08-26

**Authors:** Hongxiao Jiao, Kai Wang, Fuhua Yang, Struan F. A. Grant, Hakon Hakonarson, R. Arlen Price, Wei-Dong Li

**Affiliations:** 1 Research Center of Basic Medical Sciences, Tianjin Medical University, Tianjin, 300070, China; 2 Zilkha Neurogenetic Institute and Norris Comprehensive Cancer Center, University of Southern California, Los Angeles, CA, 90089, United States of America; 3 Center for Applied Genomics, Children’s Hospital of Philadelphia, Philadelphia, PA, 19104, United States of America; 4 Department of Pediatrics, University of Pennsylvania Perelman School of Medicine, Philadelphia, PA, 19104, United States of America; 5 Center for Neurobiology and Behavior, Department of Psychiatry, University of Pennsylvania Perelman School of Medicine, Philadelphia, PA, 19104, United States of America; Vanderbilt University Medical Center, UNITED STATES

## Abstract

Pathway-based analysis as an alternative approach can provide complementary information to single-marker genome-wide association studies (GWASs), which always ignore the epistasis and does not have sufficient power to find rare variants. In this study, using genotypes from a genome-wide association study (GWAS), pathway-based association studies were carried out by a modified Gene Set Enrichment Algorithm (GSEA) method (GenGen) for triglyceride in 1028 unrelated European-American extremely obese females (BMI≥35kg/m^2^) and normal-weight controls (BMI<25kg/m^2^), and another pathway association analysis (ICSNPathway) was also used to verify the GenGen result in the same data. The GO0009110 pathway (vitamin anabolism) was among the strongest associations with triglyceride (empirical P<0.001); the result remained significant after FDR correction (*P* = 0.022). MMAB, an obesity-related locus, included in this pathway. The ABCG1 and BCL6 gene was found in several triglyceride-related pathways (empirical *P*<0.05), which were also replicated by ICSNPathway (empirical *P*<0.05, FDR<0.05). We also performed single-marked GWAS using PLINK for TG levels (log-transformed). Significant associations were found between ASTN2 gene SNPs and plasma triglyceride levels (rs7035794, *P* = 2.24×10^−10^). Our study suggested that vitamin anabolism pathway, BCL6 gene pathways and ASTN2 gene may contribute to the genetic variation of plasma triglyceride concentrations.

## Introduction

Genome-wide association studies (GWASs) have rapidly become a powerful method for genetic studies in complex diseases. Many disease-related genes or loci have been identified with hundreds of thousands of common variants. Nevertheless, the variants identified by GWASs capture only a minor fraction of disease heritability [1, 2]. Many variants with modest associations are often ignored after multiple testing correction in GWASs[3]. Researchers can expand sample size to identify more associated loci, however, this approach will have huge cost and diminishing returns. The limitations of GWAS make it difficult to use this approach to find rare variants and epistasis [2, 4]. Pathway-based analysis is an alternative approach that detects trait-associated loci in GWAS data, which can provide complementary information to single-marker analysis, such as providing additional biological insights and highlighting new candidate genes[5, 6].

Pathway-based approaches are based on the principle that the genes always collected from the same biological or functional pathway interact with each other and constitute a network[7, 8]. Note that the context “pathway” means a gene set, rather than an interconnected biological process. Gene Set Enrichment Analysis(GSEA)[9] is a pathway-based approach, which can used to measure how much association signals are enriched in a gene set defined by known biological knowledge of genes and pathways. Wang et al. developed the GSEA-based pathway analysis, the modified GSEA method (GenGen) [10]. This approach has been successfully applied to uncover many disease-related pathways, such as the IL12/IL23 pathway associated with Crohn’s disease[11], the Vasoactive Intestinal Peptide (VIP) pathway important for Obesity[12], as well as the WNT-signaling pathway in Type2 diabetes(T2D)[13]. Unlike the above disease studies, our study focus on a quantitative trait, plasma triglyceride (TG) levels, with previous American obesity GWAS data, to examine whether TG-related gene set can be enriched.

As one of three major lipid phenotypes in the human serum, TG is a heritable trait that is a risk factor for cardiovascular disease [14, 15], insulin resistance, obesity are also characterized by increased plasma concentration of TG-rich lipoproteins[13, 14, 16]. TG level largely controlled by genetic factors, many genetic variants have been identified by GWASs. The Global Lipids Genetics Consortium(GLGC) has conducted two large-scale GWASs to identify genetic loci associated with lipid traits, and provided ~40 TG-associated genes[17, 18]. However, genetic variation at these loci explains only about 11.7% of overall TG variation within the population, corresponding to approximately 40–60% of the heritability for plasma TG levels [18–21]. Therefore, it is likely that some of genes regulating TG levels remain to be undiscovered.

In this study, we used the modified GSEA (GenGen) with extremely obese individuals and normal-weight controls, performed pathway-based association analyses to find additional susceptibility loci and pathways. In addition to GenGen, ICSNPathway (Identify candidate Causal SNPs and Pathways) [22] provides a feasible solution to bridge the gap between GWAS and disease mechanism study by generating hypothesis of SNP → gene → pathway(s). Our findings reveal several gene sets are associated with the plasma TG level, these genes may contribute to the heritability of TG.

## Results

### Single-marker association analysis for plasma TG levels

**Fig 1:**
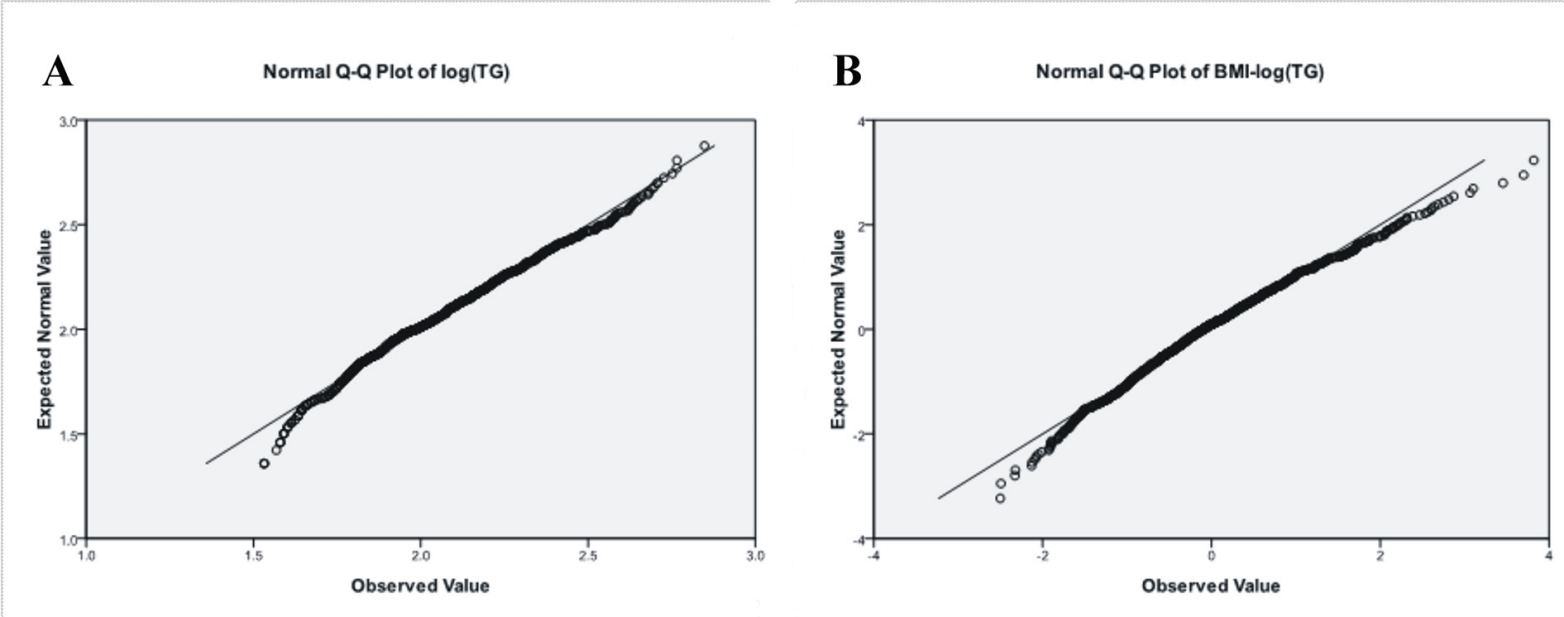
Q-Q plots of log(TG) (A) and BMI-adjusted log(TG)(B) levels in all subjects.

We examined an obesity cohort for TG levels, which previously genotyped on Illumina HumanHap550 SNP arrays for a GWAS for body weight traits[23]. TG levels were transformed by logarithms [log(TG)], and than linear regressions were carried out between log(TG) and BMI, written BMI-adjusted log(TG), we performed single-marked association analysis for the two quantitative traits, log(TG) and BMI-adjusted log(TG), in 1022 samples after extreme values (>3 SD) were deleted. Distributions of log(TG) and BMI-adjusted log(TG) levels in all samples were shown in **Table 1,** their ranges were 1.53–2.84 and -2.5–3.82, respectively. Q-Q plots showed distributions of log(TG) and BMI-adjusted log(TG) (**Fig 1**), denoted that the two phenotypes in line with normal distribution.

**Table 1:** Distribution of triglyceride levels in European-American subjects.

	N	Minimum	Maximum	Mean	SD*	Skewness	Kurtosis
Log(TG) **	1022	1.53	2.84b	2.08	0.25	0.28	-0.31
BMI-log(TG) ***	1022	-2.5	3.82	0.00	1.00	0.45	0.26

* Outliers (>±3 SD) were deleted in this study.

**Log(TG), log-transformed triglyceride levels.

***BMI-log(TG), BMI-adjusted log-transformed triglyceride levels.

**Fig 2:**
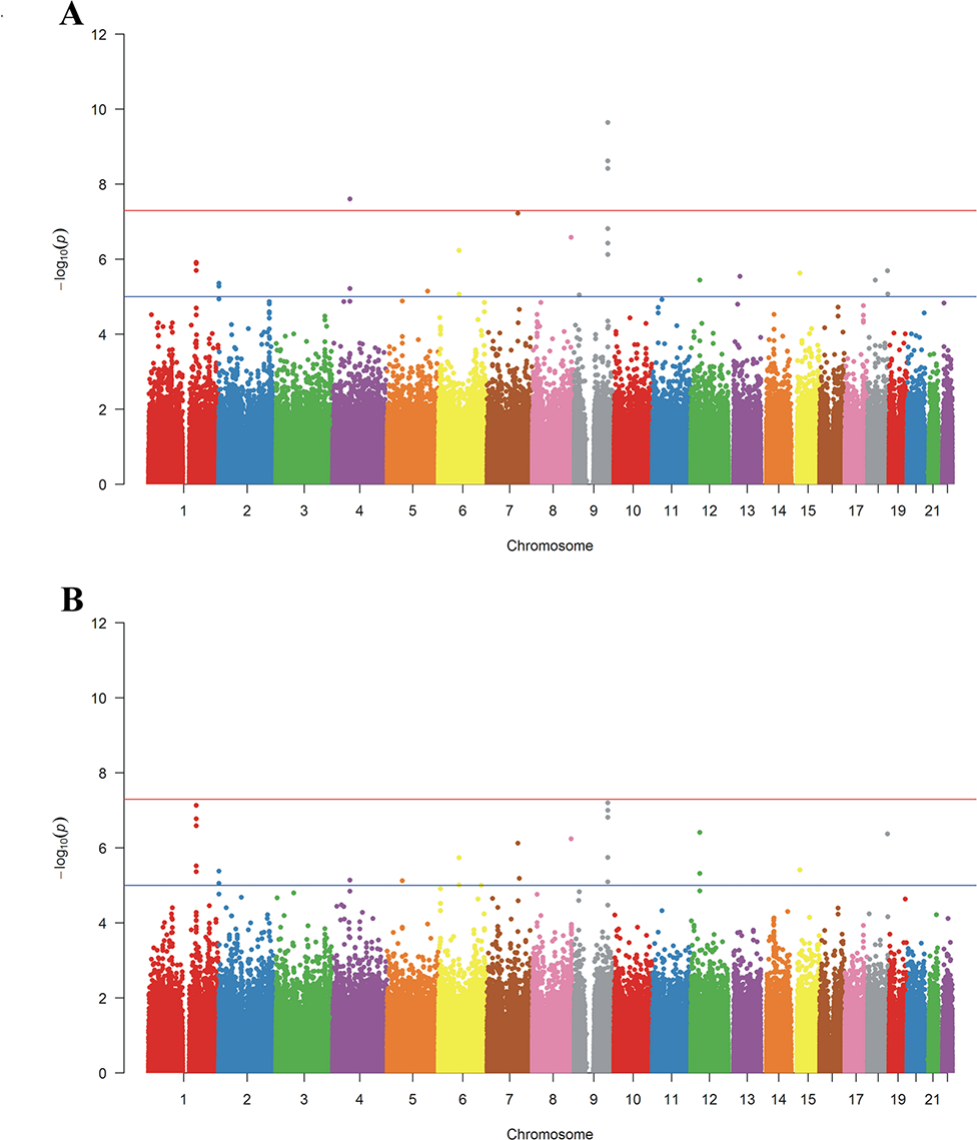
Manhattan plots for GWAS of logTG (A) and BMI-adjusted log TG (B).

Three loci on 9q33 reached genome-wide significance levels of *P* <5×10^−8^, with the most significant SNP rs7035794 (log(TG), *P* = 2.24x10^-10^; BMI-adjusted log(TG), *P* = 6.24x10^-8^) within the ASTN2 (astrotactin 2) gene. Other three SNPs in the ASTN2 gene, rs1929010, rs4091697 and rs4333683, reached *P*<1×10^−6^ **(Table 2, Fig 2**). rs2420511 (*P* = 7.38x10^-8^) and rs3817859 (*P* = 1.67x10^-7^) in C1orf112 were also associated with BMI-adjusted log(TG). Manhattan plots for GWASs of logTG and BMI-adjusted logTG were shown as (**Fig 2)**. ASTN2 is a neuronal adhesion-related gene [24], associated with Schizophrenia[24, 25], the ASTN2 association with TG was not reported before this study, although ASTN2 gene SNPs were nominally associated with total cholesterol and LDL in a GWAS for lipid phenotypes in the Framingham Heart Study[26].

**Table 2:** Quantitative association studies (PLINK) for log(TG) (*P*<10^−6^).

CHR	SNP	BP	MAF	GENE	*P*[log(TG)]	*P*[BMI-log(TG)]
9	rs7035794	118692318	0.058	ASTN2	2.24×10^−10^	6.24×10^−8^
9	rs10118539	118695771	0.077	ASTN2	2.38×10^−9^	1.00×10^−7^
9	rs4836907	118706675	0.072	ASTN2	3.79×10^−9^	1.51×10^−7^
9	rs1929010	118728015	0.068	ASTN2	7.45×10^−7^	1.80×10^−6^
9	rs4091697	118694342	0.093	ASTN2	1.52×10^−7^	8.04×10^−6^
9	rs4333683	118677830	0.074	ASTN2	3.68×10^−7^	3.33×10^−5^
1	rs2420511	168079207	0.087	C1orf112	1.20×10^−6^	7.38×10^−8^
1	rs3817859	168085689	0.089	C1orf112	1.99×10^−6^	1.67×10^−7^

No genome-wide association (*P*<5x10^-8^) was reached for binary TG **(S1 Table)**, although "top" associations (including CDKN2A/B, PABPC4L, and ABCG1) were associated with lipid and/or body weight related phenotypes[27], or had direct biology connections with cholesterol transport.

### Pathway-based analyses for plasma TG level

**Fig 3:**
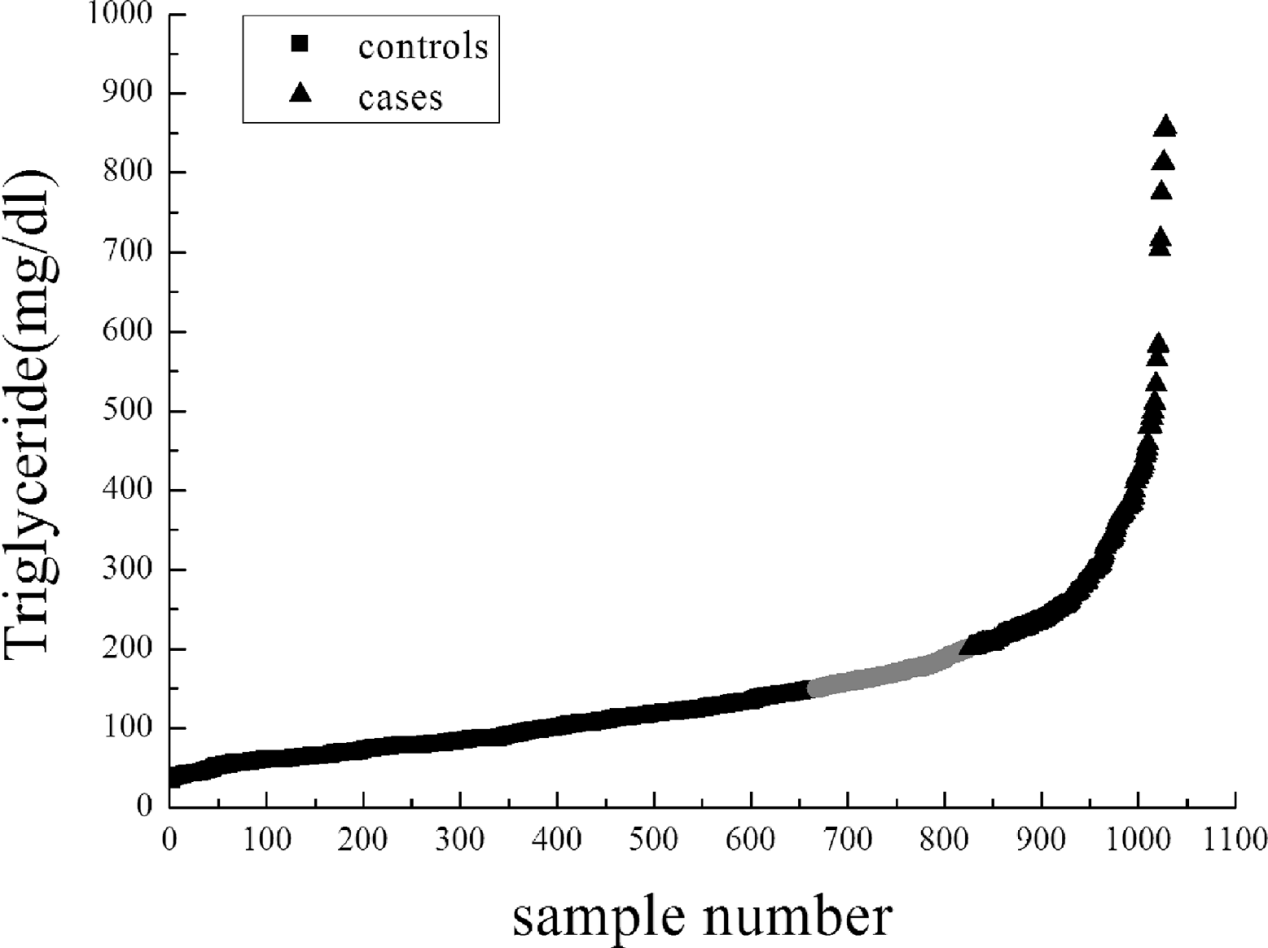
Scatter diagram of triglyceride in 1028 samples. Arranged in accordance with triglyceride levels from low to high, TG>200mg/dl as cases (N = 203) marked by black boxes, TG<150mg/dl as controls (N = 667) marked by black triangle, 150mg/dl<TG<200mg/dl as gray area were ignored in our pathway-based analysis by modified GSEA (GenGen).

To further examine novel biological pathways or collections of functionally related genes that associated with TG levels, we carried out two binary pathway-based analyses: the modified GSEA (GenGen) [6] and ICSNPathway on this GWAS data, with the same subjects and SNPs used in single-marker association analysis. In 2001, the National Cholesterol Education Program (NCEP) released recommendations on triglyceride levels that Normal triglyceride concentration is less than 150 mg/dL, borderline is 150 to 199 mg/dL, high is 200 to 499 mg/dL, and concentrations of 500 mg/dL or higher are considered very high. In this study, we defined TG levels > 200 mg/dl as “cases”, N = 203, and TG levels < 150 mg/dl as “controls”, N = 667 (**Fig 3**). Deleted the borderline value, a total of 870 samples were used for the next binary pathway association analyses. Average ages of cases and controls were 42.6±9.6 years and 42.3±8.9 years, respectively. Distributions of TG in cases and controls are shown in **Table 3.**

**Table 3:** Distribution of triglyceride levels in “cases” (TG>200mg/dL) and “controls” (TG<150mg/dL) for binary GWAS and pathway association analyses.

	N	Age	Triglycerides (mg/dL)	Maximum	Minimum	Mean	SD
Cases	203	42.6±9.6	>200	858	201	307.4	126.3
Controls	667	42.3±8.9	<150	149	34	92.4	30.0

**Fig 4:**
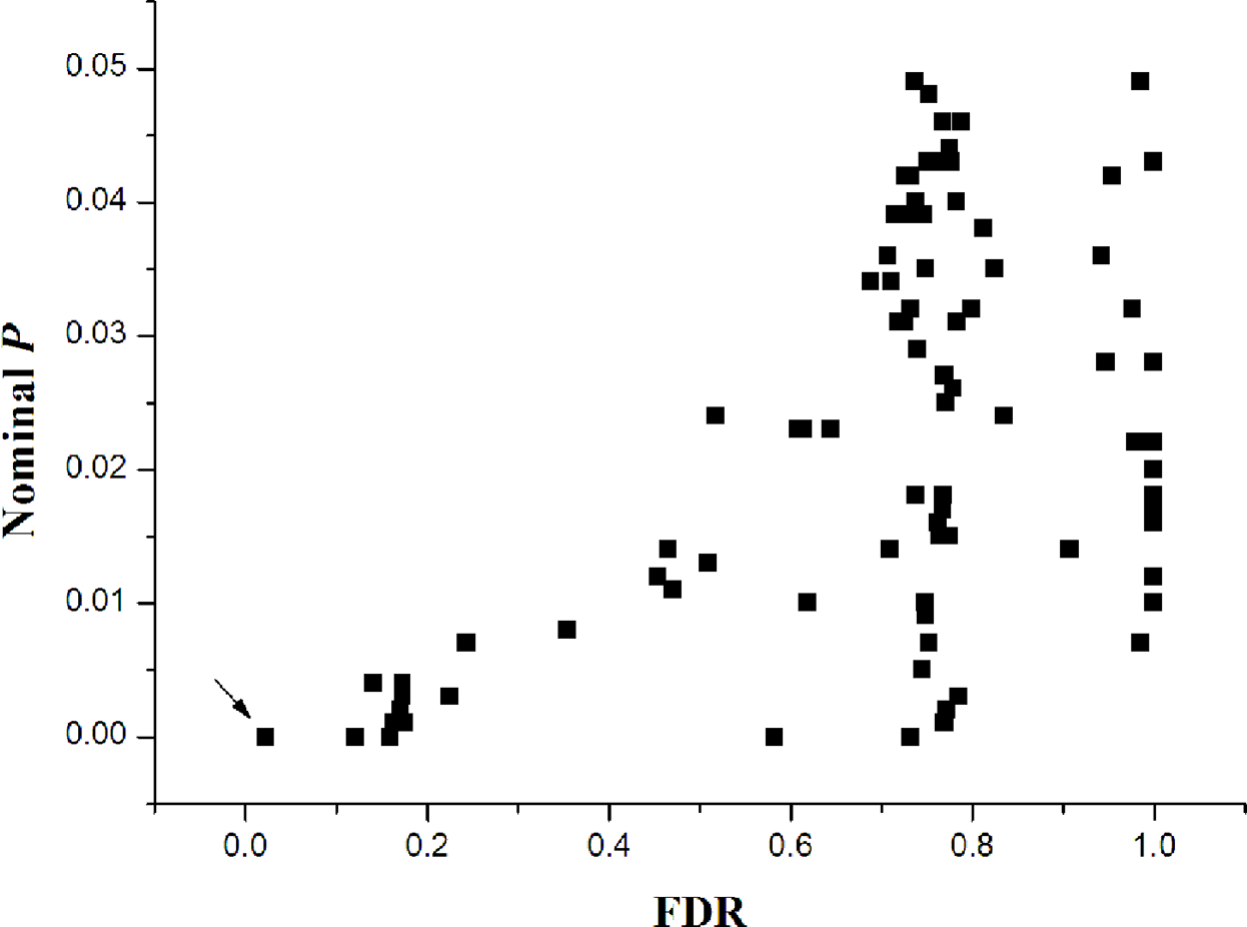
The distribution of empirical *P*-FDR for triglyceride. Empirical *P*-FDR for triglyceride related pathways (empirical *P*<0.05, denoted as “nominal” *P* values in the GenGen program) obtained by modified GSEA (GenGen), GO0009110 pathway is indicated by the arrow.

In the process of pathway analysis, the case/control labels were permuted 1000 times in our study, and for each permutation, the association test statistics for all SNP markers were recalculated, and then re-evaluation the signifcance of the pre-defined gene sets by comparing the observed test statistics with the null distribution generated by the permutations. Total 1347 gene sets were assessed, which passed the size threshold (5–200 genes), and retrieved from BioCarta, KEGG(Kyoto Encyclopedia of Genes and Genomes Pathway), and GO (Gene Ontology) databases. We found that twenty-three pathways were associated with TG-levels at an empirical *P*-value less than 0.01 in the pathway-based analysis by GenGen (**S2 Table)**. Among these pathways, GO0009110 (vitamin anabolism) showed the most significant association with TG level (empirical P<0.001) and have false discovery rate (FDR) less than 0.05 passed multiple testing corrections (FDR = 0.022), and another eight pathways have FDR less than 0.2 (**Fig 4**). There were 19 genes in the vitamin anabolism pathway, including MMAB, PNPO, PDXK, and ME1 **(S3 Table)**. The SNP rs7953794 locate in the MMAB gene yielded moderate single locus association with TG (*P* = 0.016), MMAB is a HDL-cholesterol-related gene [28]. We need to point out that the “nominal” *P* values in the GenGen program[10] were actually empirical *P* values based on permutations, therefore we used “empirical *P*” for our GenGen results.

We further used ICSNPathway to analyze pathway associations in the same data (**S4 Table)**. In this method, a SNP *P*-value file (from GWAS) and a gene set file were needed. In order to reduce the external differences between the ICSNPathway and the modified GSEA (GenGen), SNP *P*-value file was obtained from binary association analyses by PLINK, the same gene set file was used as GenGen. The results of ICSNPathway were shown as (**Fig 3)**. Nine pathways, which were among the most significant TG-associated pathways in GenGen, also reached the *P*-value<0.01 and FDR less than 0.2. Vitamin anabolism pathway had the empirical *P* = 0.002. GO0022618 (Protein-RNA complex assembly), GO0051320 (S-phase), GO0016447 (somatic recombination of immunoglobulin gene segments), and GO0045930 (negative regulation of mitotic cell cycle) were further verified to be associated with TG by ICSNPathway analyses after multiple testing corrections (nominal *P*<0.01, FDR<0.05). The BCL6 gene (B-cell CLL/lymphoma 6) was found in 6 of the top 9 TG-associated pathways, GO0051320, GO0016447, GO0045930, GO0002449 (lymphocyte mediated immunity), GO0007346 (regulation of mitotic cell cycle), and GO0002460 (adaptive immune response based on somatic recombination of immune receptors built from immunoglobulin super-family domains).

## Discussion

In the single-marker GWAS and pathway-based association studies, we identified the gene ASTN2 and the “vitamin anabolism” pathway were significantly associated with TG levels, respectively.

The neuronal adhesion-related gene ASTN2 (astrotactin 2) is expressed in the brain and has a domain structure similar to that of ASTN1; the ASTN2 + ASTN1 protein complex is important for proper cell-surface expression of ASTN1 [29]. ASTN2 is involved in neuronal adhesion and performs a key role in neural development [30, 31]. Neuronal pathways can produce an effect on insulin sensitivity [32], and contribute to insulin resistance syndrome components in humans, especially for type 2 diabetes and obesity [33]. In our study, neuronal adhesion-related gene ASTN2 was associated with plasma TG levels. A previous linkage analysis for lipid-related latent gene-expression quantitative traits with metabolic syndrome found that ASTN2 was associated with lipid levels [34].

Traditional GWASs (also called single-marker GWASs) have many limitations, including the inability to identify many “minor” genes. In our single-marker association analysis, only one gene, ASTN2, reached genome-wide significance levels of *P*<5×10^−8^. With the purpose of collecting more TG-related genes, we carried out pathway-based analysis on our GWAS data (**Table 4**), with the same subjects and SNPs used in previous single-marker GWASs. Many gene sets were associated with TG levels through the GenGen, especially the vitamin anabolism gene set.

**Table 4:** Top pathways that associated with binary TG stratification (case-control).

			GenGen		ICSNPathway		
Pathway ID	Description	Set Size	empirical *P*	FDR	Nominal *P*	FDR	Database
GO0009110	Vitamin anabolism	19	**<0.001**	**0.022**	0.002	0.157	GO
GO0022618	Protein-RNA complex assembly	43	<0.001	0.121	**<0.001**	**0.02**	GO
GO0051320	S-phase	13	<0.001	0.159	**<0.001**	**0.005**	GO
GO0002449	Lymphocyte mediated immunity	46	0.001	0.163	0.006	0.181	GO
GO0007346	Regulation of mitotic cell cycle	56	0.001	0.176	0.007	0.184	GO
GO0016447	Somatic recombination of immunoglobulin gene segments	24	0.002	0.170	**<0.001**	**0.012**	GO
GO0045930	Negative regulation of mitotic cell cycle	14	0.003	0.173	**<0.001**	**0.013**	GO
GO0005178	Integrin binding	47	0.004	0.173	0.001	0.156	GO
GO0002460	Adaptive immune response based on somatic recombination of immune receptors built from immunoglobulin superfamily domains	57	0.004	0.141	0.053	0.359	GO

GO0009110 is a vitamin anabolism pathway, the interactions among the genes exist in this pathway are related to triglyceride. MMAB [methylmalonic aciduria (cobalamin deficiency) cblB type] encodes a protein that catalyzes the conversion of vitamin B_12_ into adenosylcobalamin, an active coenzyme form of B_12_. HDL-cholesterol-associated SNP (rs7298565) is associated with higher MMAB mRNA and protein levels [28], MMAB_3U3527G→C variant also contribute to variation of HDL-cholesterol concentrations [35]. In our whole genome linkage analysis, the chromosome region 12q23-24 yielded significant linkage (LOD score = 4.08) for percentage fat [36]. The MMAB locus was only 50 kb away from the linkage peak D12S1339.

MMAB was associated with TG levels, which can be interpreted by examining its function. On the one hand, methylmalonyl-CoA mutase, as an adenosylcobalamin-dependent enzyme, can catalyze the 1,2-rearrangement of methylmalonyl-CoA to succinyl-CoA [37]. Succinyl-CoA joins the tricarboxylic acid cycle, which is the common metabolic pathway of carbohydrates, lipids, and amino acids. MMAB can affect TG levels through adenosylcobalamin and methylmalonyl-CoA mutase. Moreover, plasma B_12_ correlates inversely with homocysteine, which is an intermediate product of methionine metabolism [38]. B_12_ deficiency is a common cause of hyperhomocysteinemia [39]. Homocysteine is associated with plasma TG levels. Geoff et al. found that homocysteine-induced endoplasmic reticulum stress causes dysregulation of the endogenous sterol response pathway and leads to uptake of TG [40].

BCL6 was present in many TG-associated pathways, especially in the three pathways of the ICSNPathway analysis that passed multiple testing correction (nominal *P*<0.001, FDR<0.05). BCL6 is a transcriptional repressor which frequently disrupted by translocations in B-cell lymphomas. BCL6 can repress the transcription of BCL6 target genes, mainly involved in cell activation and differentiation, cell cycle regulation, and inflammation [41, 42]. Previous studies have demonstrated that the FOXO/BCL6/cyclin D2 pathway linked to β-cell proliferation and may therefore be considered to be associated with diabetes [43, 44]. In addition, the BCL6-SMRT and BCL6-NcoR cistromes were reported to be able to repress NF-κB-driven inflammatory immune responses [45]. Free BCL6 can attenuating inflammatory gene expression through suppressing MCP-1 in the kidney, and related to anti-inflammatory effects. For patients with severe hypertriglyceridemia, anti-inflammatory drug therapy significantly reduces TG levels [46]. Studies also have shown that elevation of TG-rich lipoprotein can induce endothelial cell inflammation [47, 48]. TG levels are usually increased in obese and diabetic individuals, and BCL6 is related to diabetes and obesity through the above description. BCL6 is associated with TG levels in our results.

Many genes were enriched in our pathway-based analysis that probably relate to TG. Several diabetes-related or inflammation-related genes are included in the TG-associated pathways, such as SMAD3 [49], TGFB1 [50], CDKN2B [13], and IL10 [51]. Needless to say, further analyses are needed to decipher the interaction among those genes.

In this study, we performed pathway-based studies using two different methods to better verify our findings. Vitamin anabolism related pathways were associated with plasma TG levels, which therefore might account for TG-related cardiovascular events. Larger sample sizes are needed in future pathway association studies in different populations to verify this result.

## Materials and Methods

### Subjects

These samples were originally collected to study obesity, and they were used to analyze associations for TG in this study. One thousand and twenty eight (1028) unrelated European Americans were chosen from an ongoing study, comprising 490 extremely obese females (BMI>35 kg/m^2^) and 538 normal-weight controls (BMI<25 kg/m^2^). All cases were obese probands, selected from obese families and trios, and unrelated normal weight controls were selected who had a current and lifetime BMI<25 kg/m^2^[23]. Clinical characteristics have been described previously [52, 53].

Note that these samples were collected originally in order to investigate obesity genes in female subjects, so we have a small fraction of males during the recruitment.

All participants gave written informed consent, and the research protocol was approved by the Institutional Review Board (IRB) on Studies Involving Human Beings at the University of Pennsylvania.

### Phenotypes

Blood samples were obtained after subjects had fasted overnight (>6 h). Plasma TG levels were measured by Quest Diagnostics (Philadelphia, PA). The standard formula, Weight (kg) divided by Height (m^2^), was used calculated Body mass index. Height was measured by a standing position using a stadiometer. Weight was measured by a scale with a maximum weight of 600 pounds (270 kg). All measurements were taken when subjects dressed in light clothing. Log-transformed TG [log(TG)] and BMI-adjusted log(TG) were the phenotypes of association analyses. For BMI-adjusted log(TG), linear regressions were carried out between log(TG) and BMI (SPSS, version 17.0), with BMI as independent variable, log(TG) as the dependent variable, and standardized residuals saved to make mean = 0 and standard deviation = 1. Threshold selected binary triglycerides were used for discrete GWAS and GenGen analyses: individuals with TG>200mg/dL were set as "cases" and those with TG<150mg/dL were used as "controls", affection statuses for those with 150mg/dL≤TG≤200mg/dL were considered as "unknown".

### Genotyping

Genomic DNA was extracted from peripheral blood using a high-salt method [54] and diluted to 10 ng/μl. Genotyping was performed for our previous GWAS for body weight traits[23]. In brief, Illumina HumanHap550 SNP arrays (Illumina, San Diego, CA), with about 550,000 SNP markers, were used to genotype DNA samples at the Center for Applied Genomics, Children’s Hospital of Philadelphia. Standard Illumina data normalization procedures and canonical genotype clustering files were used to process the genotyping signals. Hardy-Weinberg equilibrium (HWE) was tested for all SNPs in the array, SNPs with genotype frequencies that depart from HWE were deleted.

### Statistical analyses

Basic statistical descriptions were performed using SPSS 17.0. TG (log-transformed) outliers (>3 SD) were excluded from the data set, 6/1028 samples were removed, and then linear regression was carried out between log(TG) and BMI. Genome-wide quantitative association analyses were performed by PLINK 1.07 [55] for log(TG) and BMI-adjusted log(TG). SNPs with minor allele frequencies (MAF) <1% were excluded from quantitative association studies. We also performed a discrete GWAS for triglyceride using threshold-selected binary TG (TG>200mg/dL vs. TG<150mg/dL) (**Table 3**).

The pathway-based genome-wide studies were divided into two steps, firstly, we used a modified Gene Set Enrichment Analysis (GSEA) method (GenGen) developed by Wang et al. [10] to carry out binary association analysis; Secondly, we used ICSNPathway [22] to verify our pathway association results obtained by GenGen.

### Gene set enrichment analysis

This method was used performed pathway-based test for genome-wide association data. The main process was as follows. For each gene, the SNP with the highest test statistic (chi-square detection/*F*-test) among all SNPs mapped to the gene was selected to represent the gene [10]. All genes were ranked by sorting their statistic values from the largest to smallest, denoted by *r*(1),…, *r*(*N*), where *N* represents the total number of genes. For any given gene set *S* composed of *N*_*H*_ genes, an enrichment score (ES) was calculated, which was a weighted Kolmogorov-Smirnov-like running sum statistic that reflects the overrepresentation of genes within *S* at the top of the entire ranking list of genes in the genome:

$$\text{ES}(S)=\underset{1\leq j\leq N}{\text{max}}\left\{\sum_{G_{j^{*}}\in S,j^{*}\leq j}{\frac{\left|r_{\left(j^{*}\right)}\right|}{N_{R}}}^{p}-\sum_{G_{j^{*}}\notin S,j^{*}\leq j}\frac{1}{N-N_{H}}\right\},$$

Where $N_{R}={\sum_{G_{j^{*}}\in S}\left|r_{\left(j^{*}\right)}\right|}^{p}$ and *p* is a parameter that gives higher weight to genes with extremes statistic values (default *P* = 1). The phenotype label were permutated 1000 times to adjust the size of different genes. For each permutation, enrichment scores were calculated. We then calculated the normalized enrichment score (NES):

$$\text{NES}=\frac{\text{ES}(S)-\text{mean}\left[\text{ES}(S,\pi )\right]}{SD\left[\text{ES}(S,\pi )\right]}.$$

Finally, a false-discovery rate (FDR) procedure was conducted to control the fraction of expected false-positive findings:

$$\text{FDR}=\frac{\%\text{of all}\;\left(S,\pi \right)\;\text{with NES}\left(S,\pi \right)\geq NES*}{\%\text{of observed}\;S\;\text{with NES}\left(S\right)\geq NES*}.$$

where NES* denotes the normalized ES in the observed data. Approximately 520K SNPs passed the initial quality-control threshold in the analysis of GenGen(defined as minor-allele frequency > 0.01 and Hardy-Weinberg equilibrium *P*-value > 0.001), which covered 17,438 genes; 20k bp upstream and downstream of each gene was considered to be a part of the gene. We retrieved 301 annotated pathways from the BioCarta database and 212 annotated pathways from Kyoto Encyclopedia of Genes and Genomes Pathway database and constructed 2,058 gene sets on the basis of Gene Ontology (GO) annotation files, which were downloaded from the GO website. We also limited testing to those pathways that contained between 5 and 200 genes represented by markers in our GWAS database. Thus, a total of 1347 pathways were analyzed in this analysis.

### Identify Candidate Causal SNPs and Pathways (ICSNPathway)

The ICSNPathway web server implements a two-stage analysis [22]: in the first stage, the candidate causal SNPs are pre-selected according to linkage disequilibrium (LD) analysis and functional SNP annotation based on the most significant SNPs of GWAS to represent the gene; in the second stage, the biological mechanisms for the pre-selected candidate causal SNPs are annotated by the Improved Gene Set Enrichment Analysis (i-GSEA) [56], which also needs to calculate ES. SNP label permutations, however, were implemented instead of phenotype label permutations. Based on all the distributions of ESs generated by permutation, FDR is used for multiple testing corrections. In short, ICSNPathway integrates LD analysis, functional SNP annotation, and pathway-based analysis to identify candidate causal SNPs and their corresponding candidate causal pathways from GWAS data sets.

In this analysis, SNP *P*-values were obtained from binary association analyses by PLINK. We collected all SNPs for the next test. Other main parameters included: (1) LD cutoff: r^2^>0.6, (2) distance for searching LD neighborhoods: 200 kb, (3) rule of mapping SNPs to genes: 20 kb upstream and downstream of gene, and (4) gene set database: same to the GenGen gene sets files, with pathways containing <5 or >200 genes ignored. Of the 2571 pathways, 1359 passed the filtering criteria.

## Supporting Information

S1 Table Associations (*P*<1x10^-4^) for binary GWAS for triglyceride. (DOC)

S2 Table The binary pathway-based association analysis for TG by GenGen. (empirical *P*<0.05). (DOCX)

S3 Table Genes in triglyceride-related pathways. (DOC)

S4 Table The URL of the result analyzed by ICSNPathway. (DOC)
