# Supplementary material for: Pathway-Based Genome-Wide Association Studies for Plasma Triglycerides in Obese Females and Normal-Weight Controls
Source: PLoS One. 2015 Aug 26;10(8):e0134923. doi: 10.1371/journal.pone.0134923 (PMC4550433; doi:10.1371/journal.pone.0134923)
Supplement: S1 Table — (DOC) [file pone.0134923.s001.doc]

**Supplement Table S1** Associations (P<1x10^-4^) for binary GWAS for triglyceride

|  | |  | |  |  |  |  |  |  |  |
| --- | --- | --- | --- | --- | --- | --- | --- | --- | --- | --- |
| gene | chromosome | | SNP | | bp | F_A | F_U | χ^2^ | OR | *P* |
| CDKN2A/B | 9 | | rs10811581 | | 21681392 | 0.3557 | 0.2361 | 22.75 | 1.786 | 1.85 X 10^-6^ |
|  | 5 | | rs10058740 | | 113036049 | 0.1946 | 0.1059 | 22.16 | 2.041 | 2.51 X 10^-6^ |
| PABPC4L | 4 | | rs10518647 | | 135252932 | 0.0197 | 0.0007496 | 21.73 | 26.79 | 3.13 X 10^-6^ |
| ABCG1 | 21 | | rs13050646 | | 42585118 | 0.08838 | 0.03395 | 20.19 | 2.759 | 7.01 X 10^-6^ |
| FRG1 | 4 | | rs1112139 | | 190953178 | 0.165 | 0.08696 | 20.16 | 2.075 | 7.13 X 10^-6^ |
| KLF6 | 10 | | rs2129445 | | 3736775 | 0.2971 | 0.1744 | 20 | 2.001 | 7.75 X 10^-6^ |
| CDKN2A/B | 9 | | rs10965099 | | 21672759 | 0.349 | 0.2372 | 19.97 | 1.724 | 7.89 X 10^-6^ |
| NEGR1 | 1 | | rs12060170 | | 72924692 | 0.01478 | 0 | 19.81 | NA | 8.54 X 10^-6^ |
| SEC23A | 14 | | rs17108797 | | 38614950 | 0.1133 | 0.05097 | 19.75 | 2.379 | 8.83 X 10^-6^ |
| CXCL14-NEUROG1 | 5 | | rs10038760 | | 134924732 | 0.1995 | 0.1139 | 19.65 | 1.938 | 9.30 X 10^-6^ |
| PTPRZ1 | 7 | | rs10276802 | | 121256154 | 0.08374 | 0.1727 | 19.1 | 0.4379 | 1.24 X 10^-5^ |
| PDE7A | 8 | | rs7839014 | | 66897966 | 0.06965 | 0.1545 | 19.05 | 0.4096 | 1.27 X 10^-5^ |
| GALNT2 | 1 | | rs10495282 | | 228063377 | 0.01724 | 0.0007485 | 18.53 | 23.42 | 1.67 X 10^-5^ |
| FAM155A | 13 | | rs9514649 | | 106742081 | 0.5965 | 0.4749 | 18.29 | 1.635 | 1.90 X 10^-5^ |
| HAND1 | 5 | | rs1842226 | | 153860453 | 0.02217 | 0.002249 | 18.03 | 10.06 | 2.17 X 10^-5^ |
| GFI1 | 1 | | rs1325435 | | 92703851 | 0.5542 | 0.4347 | 17.87 | 1.617 | 2.36 X 10^-5^ |
| BCL11A | 2 | | rs10490066 | | 60262883 | 0.2946 | 0.1957 | 17.75 | 1.717 | 2.51 X 10^-5^ |
| RPAP2 | 1 | | rs912794 | | 92649076 | 0.5322 | 0.4137 | 17.66 | 1.612 | 2.64 X 10^-5^ |
| CSMD1 | 8 | | rs7018016 | | 4677890 | 0.1436 | 0.07496 | 17.66 | 2.069 | 2.64 X 10^-5^ |
| NEK6 | 9 | | rs2767777 | | 125998894 | 0.303 | 0.2035 | 17.57 | 1.702 | 2.77 X 10^-5^ |
| FDX1 | 11 | | rs10891123 | | 109859403 | 0.1059 | 0.04873 | 17.48 | 2.313 | 2.90 X 10^-5^ |
| CXCL14 | 5 | | rs2344483 | | 134967889 | 0.2586 | 0.1662 | 17.43 | 1.75 | 2.98 X 10^-5^ |
| ST6GAL2 | 2 | | rs6543445 | | 106810608 | 0.3366 | 0.4534 | 17.27 | 0.6118 | 3.24 X 10^-5^ |
| LINC00271 | 6 | | rs4896157 | | 135983812 | 0.4384 | 0.3261 | 17.2 | 1.614 | 3.36 X 10^-5^ |
| CXCL14 | 5 | | rs4463175 | | 134967023 | 0.2574 | 0.1657 | 17.15 | 1.746 | 3.45 X 10^-5^ |
| DCC | 18 | | rs12606713 | | 48433842 | 0.02463 | 0.08471 | 17.1 | 0.2729 | 3.54 X 10^-5^ |
| EDNRA | 4 | | rs1429107 | | 148541837 | 0.07143 | 0.02699 | 17.1 | 2.774 | 3.55 X 10^-5^ |
| SLC9A2 | 2 | | rs7567133 | | 102619560 | 0.2389 | 0.1507 | 17.08 | 1.769 | 3.59 X 10^-5^ |
| RGPD3 | 2 | | rs6753897 | | 106569244 | 0.07389 | 0.1538 | 17 | 0.4388 | 3.73 X 10^-5^ |
| RPAP2-GFI1 | 1 | | rs4414050 | | 92663125 | 0.5322 | 0.4159 | 16.98 | 1.598 | 3.78 X 10^-5^ |
| LOC150935 | 2 | | rs12614692 | | 200116000 | 0.1847 | 0.1074 | 16.98 | 1.884 | 3.78 X 10^-5^ |
| GPR56 | 16 | | rs17325839 | | 56234402 | 0.2635 | 0.1717 | 16.83 | 1.727 | 4.08 X 10^-5^ |
| KITLG | 12 | | rs7310282 | | 87671300 | 0.03465 | 0.007485 | 16.83 | 4.76 | 4.08 X 10^-5^ |
|  | 2 | | rs728849 | | 83650688 | 0.5764 | 0.4601 | 16.82 | 1.596 | 4.10 X 10^-5^ |
| CDYL | 6 | | rs6907963 | | 4903481 | 0.3317 | 0.4466 | 16.81 | 0.6149 | 4.14 X 10^-5^ |
| PDE7A | 8 | | rs6472228 | | 66918903 | 0.07882 | 0.1596 | 16.8 | 0.4504 | 4.16 X 10^-5^ |
| LOC150935 | 2 | | rs7603546 | | 200121000 | 0.5049 | 0.3904 | 16.79 | 1.593 | 4.18 X 10^-5^ |
| GPR56 | 16 | | rs12103178 | | 56226229 | 0.5297 | 0.4144 | 16.71 | 1.592 | 4.35 X 10^-5^ |
| RAB38 | 11 | | rs4479012 | | 87363111 | 0.1709 | 0.09633 | 16.67 | 1.934 | 4.44 X 10^-5^ |
| FHIT | 3 | | rs9813516 | | 60268044 | 0.3317 | 0.2301 | 16.61 | 1.66 | 4.59 X 10^-5^ |
| CR627240 | 9 | | rs12346101 | | 23650510 | 0.01232 | 0 | 16.5 | NA | 4.86 X 10^-5^ |
| ABHD4 | 14 | | rs1242933 | | 22149570 | 0.5833 | 0.4671 | 16.43 | 1.597 | 5.05 X 10^-5^ |
| MYO16-IRS2 | 13 | | rs1328247 | | 108917530 | 0.2709 | 0.1794 | 16.24 | 1.7 | 5.60 X 10^-5^ |
| RELN | 7 | | rs2299381 | | 103205235 | 0.5371 | 0.4233 | 16.22 | 1.581 | 5.64 X 10^-5^ |
| CDH6 | 5 | | rs1009841 | | 29512926 | 0.4005 | 0.2941 | 16.07 | 1.603 | 6.09 X 10^-5^ |
| GPR56 | 16 | | rs3916059 | | 56229407 | 0.5396 | 0.4264 | 16.03 | 1.576 | 6.25 X 10^-5^ |
|  | 19 | | rs10412222 | | 36978006 | 0.02475 | 0.003748 | 15.99 | 6.746 | 6.37 X 10^-5^ |
| RELN | 7 | | rs2299383 | | 103206082 | 0.5348 | 0.4217 | 15.97 | 1.577 | 6.43 X 10^-5^ |
| PLEKHA7 | 11 | | rs10832689 | | 16802889 | 0.3871 | 0.2786 | 15.94 | 1.636 | 6.53 X 10^-5^ |
| TOM1L1 | 17 | | rs8064660 | | 50330822 | 0.1675 | 0.09595 | 15.94 | 1.896 | 6.55 X 10^-5^ |
| FAM155A | 13 | | rs4771547 | | 106483849 | 0.5714 | 0.4586 | 15.85 | 1.574 | 6.85 X 10^-5^ |
| ZNF683-LIN28A | 1 | | rs12722898 | | 26593874 | 0.4926 | 0.382 | 15.77 | 1.571 | 7.15 X 10^-5^ |
|  | 10 | | rs10881857 | | 82385295 | 0.2764 | 0.1845 | 15.73 | 1.688 | 7.29 X 10^-5^ |
| AK091889 | 4 | | rs4453935 | | 13517883 | 0.09606 | 0.04423 | 15.73 | 2.296 | 7.29 X 10^-5^ |
| PRKCE | 2 | | rs935653 | | 45942048 | 0.08374 | 0.1627 | 15.71 | 0.4705 | 7.40 X 10^-5^ |
|  | 22 | | rs4823934 | | 48184015 | 0.3128 | 0.217 | 15.7 | 1.643 | 7.43 X 10^-5^ |
| CDKN2A/B | 9 | | rs7860632 | | 21673289 | 0.06897 | 0.1432 | 15.58 | 0.4433 | 7.90 X 10^-5^ |
|  | 12 | | rs1872539 | | 127355805 | 0.2833 | 0.3907 | 15.5 | 0.6163 | 8.24 X 10^-5^ |
| ABHD4 | 14 | | rs1242927 | | 22146746 | 0.4802 | 0.3709 | 15.49 | 1.567 | 8.31 X 10^-5^ |
| SCFD2 | 4 | | rs10012324 | | 53573909 | 0.4134 | 0.3081 | 15.48 | 1.582 | 8.33 X 10^-5^ |
| HEBP2-NHSL1 | 6 | | rs7746523 | | 138781272 | 0.0665 | 0.02553 | 15.43 | 2.72 | 8.57 X 10^-5^ |
| C1orf146 | 1 | | rs3131820 | | 92452959 | 0.5322 | 0.4213 | 15.42 | 1.563 | 8.59 X 10^-5^ |
| LOC100506368 | 11 | | rs11234894 | | 86357222 | 0.4751 | 0.3662 | 15.38 | 1.567 | 8.77 X 10^-5^ |
| LINC00520 | 14 | | rs10144952 | | 55412322 | 0.1478 | 0.08195 | 15.34 | 1.943 | 8.98 X 10^-5^ |
| GALNT2 | 1 | | rs2026539 | | 228056618 | 0.01478 | 0.0007485 | 15.31 | 20.02 | 9.10 X 10^-5^ |
| IL1R2 | 2 | | rs13414479 | | 101959201 | 0.01478 | 0.0007485 | 15.31 | 20.02 | 9.10 X 10^-5^ |
| PTBP3-MIR3134 | 9 | | rs7869523 | | 114022370 | 0.5448 | 0.4338 | 15.3 | 1.562 | 9.18 X 10^-5^ |
| NXPE1 | 11 | | rs7950370 | | 113916553 | 0.1849 | 0.1093 | 15.23 | 1.849 | 9.51 X 10^-5^ |
| PCLO | 7 | | rs4732493 | | 82527837 | 0.2389 | 0.1552 | 15.14 | 1.709 | 9.99 X 10^-5^ |

F_U: allele frequencies in unaffected individuals

F_A: allele frequencies in affected individuals
