# Supplementary material for: Pathway-Based Genome-Wide Association Studies for Plasma Triglycerides in Obese Females and Normal-Weight Controls
Source: PLoS One. 2015 Aug 26;10(8):e0134923. doi: 10.1371/journal.pone.0134923 (PMC4550433; doi:10.1371/journal.pone.0134923)
Supplement: S2 Table — (DOCX) [file pone.0134923.s002.docx]

**Supplement Table S2** **The binary pathway-based association analysis for TG by GenGen. (nominal *P*<0.05)**

| *Pathway ID* | *Set size* | *Nominal P* | *FDR* | *FWER* | *Database* |
| --- | --- | --- | --- | --- | --- |
| GO0009110 | 19 | 0 | 0.022 | 0.021 | GO |
| GO0022618 | 43 | 0 | 0.121 | 0.266 | GO |
| GO0051320 | 13 | 0 | 0.159 | 0.497 | GO |
| GO0019218 | 11 | 0 | 0.582 | 1 | GO |
| GO0032720 | 6 | 0 | 0.732 | 1 | GO |
| GO0002449 | 46 | 0.001 | 0.163 | 0.246 | GO |
| GO0007346 | 56 | 0.001 | 0.176 | 0.456 | GO |
| GO0030512 | 5 | 0.001 | 0.77 | 1 | GO |
| GO0016447 | 24 | 0.002 | 0.17 | 0.691 | GO |
| GO0007020 | 7 | 0.002 | 0.773 | 1 | GO |
| GO0045930 | 14 | 0.003 | 0.173 | 0.738 | GO |
| GO0002429 | 24 | 0.003 | 0.225 | 0.855 | GO |
| GO0046890 | 13 | 0.003 | 0.785 | 1 | GO |
| GO0002460 | 57 | 0.004 | 0.141 | 0.522 | GO |
| GO0005178 | 47 | 0.004 | 0.173 | 0.645 | GO |
| GO0003995 | 15 | 0.005 | 0.745 | 1 | GO |
| GO0007507 | 140 | 0.007 | 0.244 | 0.9 | GO |
| GO0045948 | 5 | 0.007 | 0.753 | 1 | GO |
| hsa00760 | 21 | 0.007 | 0.986 | 0.537 | KEGG |
| GO0002377 | 25 | 0.008 | 0.354 | 0.965 | GO |
| GO0048194 | 10 | 0.009 | 0.749 | 1 | GO |
| GO0048219 | 10 | 0.009 | 0.749 | 1 | GO |
| GO0048220 | 10 | 0.009 | 0.749 | 1 | GO |
| GO0002208 | 20 | 0.01 | 0.619 | 1 | GO |
| GO0016783 | 5 | 0.01 | 0.748 | 1 | GO |
| hsa05060 | 16 | 0.01 | 1 | 0.931 | KEGG |
| GO0002764 | 28 | 0.011 | 0.471 | 0.994 | GO |
| GO0051436 | 59 | 0.012 | 0.454 | 0.994 | GO |
| malatexPathway | 7 | 0.012 | 1 | 1 | Biocarta |
| GO0051187 | 30 | 0.013 | 0.509 | 0.997 | GO |
| GO0032990 | 163 | 0.014 | 0.466 | 0.99 | GO |
| GO0006891 | 18 | 0.014 | 0.71 | 1 | GO |
| erbB4pathway | 7 | 0.014 | 0.907 | 0.813 | Biocarta |
| GO0014003 | 8 | 0.015 | 0.775 | 1 | GO |
| GO0043425 | 5 | 0.015 | 0.765 | 1 | GO |
| GO0048536 | 5 | 0.016 | 0.762 | 1 | GO |
| integrinPathway | 38 | 0.016 | 1 | 0.666 | Biocarta |
| GO0048199 | 12 | 0.017 | 0.768 | 1 | GO |
| hsa04650 | 122 | 0.017 | 1 | 0.806 | KEGG |
| GO0046165 | 23 | 0.018 | 0.769 | 1 | GO |
| GO0042135 | 6 | 0.018 | 0.739 | 1 | GO |
| etsPathway | 18 | 0.018 | 1 | 0.77 | Biocarta |
| il3Pathway | 14 | 0.02 | 1 | 1 | Biocarta |
| mcmPathway | 17 | 0.022 | 1 | 1 | Biocarta |
| hsa00030 | 22 | 0.022 | 0.979 | 0.95 | KEGG |
| GO0006839 | 49 | 0.023 | 0.608 | 1 | GO |
| GO0033261 | 13 | 0.023 | 0.644 | 1 | GO |
| GO0009306 | 35 | 0.023 | 0.615 | 1 | GO |
| GO0006801 | 19 | 0.024 | 0.517 | 0.997 | GO |
| GO0006890 | 18 | 0.024 | 0.835 | 1 | GO |
| GO0002020 | 10 | 0.025 | 0.771 | 1 | GO |
| GO0032715 | 5 | 0.026 | 0.779 | 1 | GO |
| GO0042147 | 9 | 0.027 | 0.77 | 1 | GO |
| glycolysisPathway | 9 | 0.028 | 1 | 1 | Biocarta |
| hsa04330 | 45 | 0.028 | 0.947 | 0.975 | KEGG |
| GO0016646 | 14 | 0.029 | 0.74 | 1 | GO |
| GO0042110 | 124 | 0.031 | 0.725 | 1 | GO |
| GO0008188 | 36 | 0.031 | 0.719 | 1 | GO |
| GO0033365 | 6 | 0.031 | 0.784 | 1 | GO |
| GO0001736 | 5 | 0.032 | 0.733 | 1 | GO |
| ifnaPathway | 9 | 0.032 | 0.976 | 1 | Biocarta |
| hsa00512 | 29 | 0.032 | 0.8 | 0.975 | KEGG |
| GO0050982 | 9 | 0.034 | 0.711 | 1 | GO |
| GO0007613 | 17 | 0.034 | 0.688 | 1 | GO |
| GO0010608 | 65 | 0.035 | 0.749 | 1 | GO |
| GO0046889 | 5 | 0.035 | 0.825 | 1 | GO |
| GO0019965 | 32 | 0.036 | 0.708 | 1 | GO |
| etcPathway | 9 | 0.036 | 0.942 | 1 | Biocarta |
| GO0046824 | 7 | 0.038 | 0.812 | 1 | GO |
| GO0051325 | 31 | 0.039 | 0.725 | 1 | GO |
| GO0051537 | 13 | 0.039 | 0.716 | 1 | GO |
| GO0051437 | 62 | 0.039 | 0.747 | 1 | GO |
| GO0015671 | 12 | 0.039 | 0.744 | 1 | GO |
| GO0016628 | 19 | 0.04 | 0.739 | 1 | GO |
| GO0046912 | 5 | 0.04 | 0.783 | 1 | GO |
| GO0006767 | 42 | 0.042 | 0.733 | 1 | GO |
| GO0051149 | 5 | 0.042 | 0.727 | 1 | GO |
| hsa00020 | 26 | 0.042 | 0.955 | 0.994 | KEGG |
| GO0006417 | 63 | 0.043 | 0.751 | 1 | GO |
| GO0001953 | 7 | 0.043 | 0.768 | 1 | GO |
| GO0031023 | 7 | 0.043 | 0.777 | 1 | GO |
| s1pPathway | 6 | 0.043 | 1 | 1 | Biocarta |
| GO0002483 | 6 | 0.044 | 0.775 | 1 | GO |
| GO0006929 | 7 | 0.046 | 0.768 | 1 | GO |
| GO0051324 | 8 | 0.046 | 0.788 | 1 | GO |
| GO0045727 | 7 | 0.048 | 0.753 | 1 | GO |
| GO0009743 | 15 | 0.049 | 0.737 | 1 | GO |
| hsa05320 | 49 | 0.049 | 0.985 | 0.998 | KEGG |
