# Supplementary material for: Pathway-Based Genome-Wide Association Studies for Plasma Triglycerides in Obese Females and Normal-Weight Controls
Source: PLoS One. 2015 Aug 26;10(8):e0134923. doi: 10.1371/journal.pone.0134923 (PMC4550433; doi:10.1371/journal.pone.0134923)
Supplement: S3 Table — (DOC) [file pone.0134923.s003.doc]

**Supplement Table S3 Genes in triglyceride-related pathways**

| *GO0009110* | *GO0051320* | *GO0016447* | *GO0051320* |
| --- | --- | --- | --- |
| AKR1A1 | APBB1 | BCL6 | APBB1 |
| CYP27B1 | APBB2 | CD28 | APBB2 |
| DHRS9 | BCL6 | CD40LG | BCL6 |
| G6PD | BRCA2 | ERCC1 | BRCA2 |
| ITGB1BP3 | CDK2AP1 | EXO1 | CDK2AP1 |
| KYNU | CDT1 | FOXP1 | CDT1 |
| LOC554235 | CHMP1A | HSPD1 | CHMP1A |
| ME1 | DDX11 | IFNG | DDX11 |
| MMAB | GAS1 | IL10 | GAS1 |
| NADSYN1 | MYO16 | IL2 | MYO16 |
| NAMPT | POLA1 | IL27RA | POLA1 |
|  | POLD1 | IL4 | POLD1 |
| NMNAT1 | RCC1 | LIG4 | RCC1 |
| NMNAT2 | TIPIN | MLH1 | TIPIN |
| NMNAT3 |  | MSH2 |  |
| NRK1 |  | MSH3 |  |
| PDXK |  | MSH6 |  |
| PNPO |  | NBN |  |
| PSAT1 |  | STAT6 |  |
| QPRT |  | SWAP70 |  |
| RFK |  | TBX21 |  |
|  |  | TGFB1 |  |
|  |  | TNFSF13 |  |
|  |  | XRCC4 |  |

**Supplement Table S3 Genes in triglyceride-related pathways (continued)**

| GO0007346 | GO0022618 | GO0002460 | GO0005178 | GO0002449 |
| --- | --- | --- | --- | --- |
| AFAP1L2 | ASCC3L1 | BCL10 | ACTN1 | BCL10 |
| APBB1 | ATXN2 | BCL3 | ACTN2 | BCL6 |
| APBB2 | BMS1 | BCL6 | ACTN3 | C3 |
| ATM | CRNKL1 | C3 | ACTN4 | CADM1 |
| BCL6 | CUGBP1 | CD27 | ADAM10 | CD27 |
| BLM | CUGBP2 | CD28 | ADAM11 | CD28 |
| BRCA2 | DDX1 | CD40LG | ADAM2 | CD40LG |
| BRSK1 | DDX20 | CD74 | ADAM22 | CD74 |
| BUB1 | DDX23 | CD80 | ADAM23 | CD8A |
| BUB1B | DICER1 | CD86 | ADAM9 | CRTAM |
| BUB3 | EIF2A | CD8A | ADAMDEC1 | DMA |
| C9orf127 | EIF2AK1 | DMA | ADAMTS13 | ERCC1 |
| CCNA2 | EIF2AK3 | EBI3 | ADAMTS5 | EXO1 |
| CDC123 | EIF2S1 | ERCC1 | ADAMTS8 | FCER1A |
| CDC25C | EIF6 | EXO1 | ANGPTL3 | FCER1G |
| CDC6 | FUSIP1 | FCER1A | COL16A1 | FOXJ1 |
| CDK10 | GEMIN6 | FCER1G | COL3A1 | FOXP3 |
| CDK2 | MBNL | FOXJ1 | COL4A3 | GNL1 |
| CDKN2B | NIP7 | FOXP3 | CTGF | HLA-DMA |
| CDT1 | NPM1 | GNL1 | DMP1 | HSPD1 |
| CENPF | PRPF31 | HLA-DMA | DST | IFNG |
| CHFR | PRPF6 | HSPD1 | ECM2 | IL10 |
| CHMP1A | RPS14 | IFNG | EDIL3 | IL12A |
| DGKZ | SF1 | IL10 | EGFL6 | IL12B |
| DLG1 | SF3A1 | IL12B | ERBB2IP | IL2 |
| FBXO5 | SF3A2 | IL18 | FBLN5 | IL27RA |
| FOXG1 | SF3A3 | IL18BP | FCER2 | IL4 |
| FOXO4 | SFRS2IP | IL2 | GPNMB | IRF7 |
| GAS1 | SFRS5 | IL27 | ICAM2 | LIG4 |
| GML | SFRS6 | IL27RA | ICAM3 | LY9 |
| GTSE1 | SFRS8 | IL4 | ICAM4 | MALT1 |
| HUS1 | SFRS9 | IL6 | ITGA5 | MAP3K7 |
| KNTC1 | SIP1 | IRF7 | ITGB1 | MLH1 |
| MAD1L1 | SLU7 | LIG4 | ITGB3 | MSH2 |
| MAD2L1 | SMN1 | LY9 | ITGB5 | MSH6 |
| MAD2L2 | SMNDC1 | MALT1 | ITGB6 | MYD88 |
| MAEA | SNRPD1 | MAP3K7 | LAMA5 | NBN |
| MDM2 | SNRPD2 | MLH1 | LTBP4 | PRKCD |
| MTBP | SNRPE | MSH2 | NISCH | RBM24 |
| MYO16 | SNRPG | MSH6 | NPNT | RBM4 |
| NAE1 | SRPK2 | MYD88 | SYK | SLA2 |
| NBN | SRRP35 | NBN | TGFBI | SPN |
| RAD17 | TNF | NFKB2 | THY1 | STAT6 |
| RBM38 | TSR1 | PRKCD | TIMP2 | SWAP70 |
| RCC1 | TXNL4A | RARA | TNN | TBX21 |
| RINT1 | USP39 | RBM24 | TNXB | TGFB1 |
| RPRM | VCX | RBM4 | VCAM1 | TLR8 |
| SNF1LK |  | RELB | VWF | TNFSF13 |
| TERF1 |  | SLA2 |  | TRAF2 |
| TGFB1 |  | SOCS5 |  | TRAF6 |
| TP53 |  | SPN |  |  |
| TRIAP1 |  | STAT6 |  |  |
| TRRAP |  | SWAP70 |  |  |
| TTK |  | TBX21 |  |  |
| ZW10 |  | TGFB1 |  |  |
| ZWILCH |  | TLR4 |  |  |
| ZWINT |  | TLR6 |  |  |
| p53 |  | TLR8 |  |  |
|  |  | TNFSF13 |  |  |
|  |  | TNFSF13B |  |  |
|  |  | TRAF2 |  |  |
|  |  | TRAF6 |  |  |
