# Supplementary material for: Pathway-Based Genome-Wide Association Studies for Plasma Triglycerides in Obese Females and Normal-Weight Controls
Source: PLoS One. 2015 Aug 26;10(8):e0134923. doi: 10.1371/journal.pone.0134923 (PMC4550433; doi:10.1371/journal.pone.0134923)
Supplement: S4 Table — (DOC) [file pone.0134923.s004.doc]

**Table S4** The URL of the result analyzed by ICSNPathway

| URL |
| --- |
| http://ICSNPathway.psych.ac.cn/getResult.do?tag=2BB7B996DD274CC7B26BEA1C0DD426D5_1410344663752 |
| http://ICSNPathway.psych.ac.cn/getResult.do?tag=2BB7B996DD274CC7B26BEA1C0DD426D5_1410340506114 |
| http://ICSNPathway.psych.ac.cn/getResult.do?tag=2BB7B996DD274CC7B26BEA1C0DD426D5_1410336876498 |
